# Non-invasive prognostic biomarker of lung cancer patients with brain metastases: Recurrence quantification analysis of heart rate variability

**DOI:** 10.3389/fphys.2022.987835

**Published:** 2022-09-06

**Authors:** Guangqiao Li, Shuang Wu, Huan Zhao, Weizheng Guan, Yufu Zhou, Bo Shi

**Affiliations:** ^1^ School of Medical Imaging, Bengbu Medical College, Bengbu, China; ^2^ Anhui Key Laboratory of Computational Medicine and Intelligent Health, Bengbu Medical College, Bengbu, China; ^3^ Department of Radiation Oncology, First Affiliated Hospital, Bengbu Medical College, Bengbu, China

**Keywords:** heart rate variability, autonomic nervous system, lung cancer, brain metastasis, nonlinear dynamics, recurrence quantification analysis

## Abstract

**Background:** It has previously been shown that the time-domain characteristic of heart rate variability (HRV) is an independent prognostic factor for lung cancer patients with brain metastasis (LCBM). However, it is unclear whether the nonlinear dynamic features contained in HRV are associated with prognosis in patients with LCBM. Recurrence quantification analysis (RQA) is a common nonlinear method used to characterize the complexity of heartbeat interval time series. This study was aimed to explore the association between HRV RQA parameters and prognosis in LCBM patients.

**Methods:** Fifty-six LCBM patients from the Department of Radiation Oncology, the First Affiliated Hospital of Bengbu Medical College, were enrolled in this study. Five-minute ECG data were collected by a mini-ECG recorder before the first brain radiotherapy, and then heartbeat interval time series were extracted for RQA. The main parameters included the mean diagonal line length (Lmean), maximal diagonal line length (Lmax), percent of recurrence (REC), determinism (DET) and Shannon entropy (ShanEn). Patients were followed up (the average follow-up time was 19.2 months, a total of 37 patients died), and the relationships between the RQA parameters and survival of LCBM patients were evaluated by survival analysis.

**Results:** The univariate analysis showed that an Lmax of >376 beats portended worse survival in LCBM patients. Multivariate Cox regression analysis revealed that the Lmax was still an independent prognostic factor for patients with LCBM after adjusting for confounders such as the Karnofsky performance status (KPS) (HR = 0.318, 95% CI: 0.151–0.669, *p* = 0.003).

**Conclusion:** Reduced heartbeat complexity indicates a shorter survival time in patients with LCBM. As a non-invasive biomarker, RQA has the potential for application in evaluating the prognosis of LCBM patients.

## Introduction

Brain metastasis (BM) arises from the spread of malignant tumors from any body part to the brain. It occurs in up to 40% of cancer patients (Singh et al., 2018), among which lung cancer (LC) is the most common (Goldberg et al., 2015), accounting for approximately 50% of the primary tumors of BM (Yousefi et al., 2017). Approximately 10–25% of LC patients present with BM at initial diagnosis, and 40–50% of LC patients will develop BM during the disease course (Huang and Ouyang, 2013; Peters et al., 2016). BM has become the foremost cause of death in LC patients (Dragoj et al., 2017; Yousefi et al., 2017; Rybarczyk-Kasiuchnicz et al., 2021). The natural survival time of LC patients with BM (LCBM) is only approximately 2 months (Owonikoko et al., 2014). Accurate individualized treatment has contributed to an improvement in the survival of patients, and powerful prognostic evaluation can assist clinicians in formulating relevant treatment strategies for LCBM patients (Hu et al., 2010). However, the existing traditional prediction methods, such as prognostic scoring systems for LCBM patients, are prone to biases in the evaluation process, thus reducing their predictive effect (Chiang et al., 2010). Therefore, it becomes imperative to find an objective and reliable prognostic index.

The imbalance of the autonomic nervous system (ANS) is closely related to the occurrence and development of many diseases. Decreased vagus nerve (VN) activity can increase the risk of cancer progression (Gidron et al., 2005), which is mainly due to the negative correlation between vagal activity and three basic mechanisms of tumorigenesis (local oxidative stress and DNA damage, inflammatory response and excessive sympathetic activity) (Gallowitsch-Puerta and Pavlov, 2007; Hayes et al., 2020; Zahalka and Frenette, 2020). Heart rate variability (HRV) is a noninvasive biomarker to characterize vagal activity (Lombardi and Stein, 2011). Lower HRV often portends poor vagal activity (Kuo et al., 2005). The methods for quantitative analysis of HRV are mainly based on linear methods in the time and frequency domains and nonlinear methods. Existing studies on the relationship between the time- and frequency-domain parameters of HRV and the prognosis of many kinds of malignant tumors, such as liver cancer (Chiang et al., 2010), pancreatic cancer (De Couck et al., 2016), BM (Wang et al., 2013; Wang et al., 2021), colorectal cancer (Strous et al., 2020) and LCBM (Wu et al., 2022), have shown that a low level of linear HRV is associated with poor prognosis of cancer patients, which is of great significance for the formulation of treatment strategies for cancer patients.

However, due to the interaction of multiple physiological mechanisms, the heartbeat interval time series is a dynamic system with nonstationary and highly complex features. The simple linear analysis method is not enough to evaluate the complex characteristics generated by heartbeats. Nonlinear analysis can supplement the physiological information that cannot be detected by linear analysis to assess better the complex changes in heart rate dynamics (Lipsitz, 1995; Trimer et al., 2014; Shi et al., 2019). It has been proven that nonlinear HRV can provide a new idea for the progression and prognostic evaluation of cancer. For example, Bettermann et al. (2001) showed that the complexity of HRV in breast cancer patients was lower than that in age-matched healthy women and compared with breast cancer patients without metastasis, breast cancer patients with metastasis had lower complexity. In a preliminary study on gastric cancer, Shi et al. (2019) found that nonlinear HRV parameters such as detrended fluctuation analysis (DFA), heart rate asymmetry and entropy were significantly correlated with tumor markers, and the perturbations of HRV nonlinear dynamic patterns predicted increased gastric cancer severity.

Most nonlinear analysis methods, such as DFA, fractal dimension and Lyapunov exponents, require a long or even stationary time series (Calderón-Juárez et al., 2020). Recurrence quantification analysis (RQA) is a nonlinear analysis method suitable for high noise and non-stationary short-term time series. It can effectively detect the transformation of system dynamics from time series (Marwan et al., 2007) and is also suitable for heartbeat interval time series (Marwan et al., 2002). Previous studies have shown that compared with normal subjects, the RQA indices of patients such as diabetes mellitus and paroxysmal atrial fibrillation (PAF) are significantly lower (Mohebbi and Ghassemian, 2011; Acharya et al., 2013). However, there are no studies of RQA indices prognosis for LCBM patients. Therefore, the purpose of this study is to explore the relationship between the survival and RPA parameters in LCBM patients. We hypothesized that the decrease in heartbeat complexity characterized by the RQA indicates a poor prognosis in patients with LCBM.

## Materials and methods

### Subjects

Fifty-six patients with LCBM were enrolled in this study from the Department of Radiation Oncology, the First Affiliated Hospital of Bengbu Medical College (Bengbu, Anhui, China), between October 2019 and April 2021. The patient inclusion criteria were as follows: 1) LC diagnosed by histopathology or cytopathology; 2) BM confirmed by imaging examination, such as enhanced computed tomography (CT) or magnetic resonance imaging (MRI); and 3) received brain radiotherapy for the first time. The following patients were excluded: those with 1) pacemakers; 2) severe dysfunction of important organs, such as the heart, liver and kidney; 3) previous use of drugs affecting HRV; 4) previous brain radiation or surgery; or 5) incomplete medical records. After being informed of the detailed procedures and possible risks of the experiment, all participants voluntarily participated and signed informed consent. This study was approved by the Institutional Review Board (IRB) of the First Affiliated Hospital of Bengbu Medical College (IRB number: 2019KY031). The research process is strictly in accordance with the Declaration of Helsinki.

### Data collection

The ECG data of patients with LCBM before the first brain radiotherapy were recorded by a single-lead mini-ECG recorder (HeaLink-R211B; HeaLink Ltd., Bengbu, China), and the collection time was 5 min. Patients should relax in advance and be placed in a supine position, be asked to breathe smoothly and not speak during the ECG collection process. The whole process was carried out in a room where the temperature was maintained at 23 ± 1°C, and there was no noise disturbance.

We collected the basic data of LCBM patients, including sex, age, body mass index (BMI), Karnofsky performance status (KPS), pathological types of LC, primary tumor status, the presence of extracranial metastasis, number of BM and whether systemic therapy was performed after BM. In addition, we also calculated the mean heart rate (mean HR) and respiration rate (RR) of each patient by ECG data.

### Heart rate variability analysis

Recursion is one of the basic properties of dynamical systems, which means that some states of the system have similar or repetitive characteristics in a specific time frame. The recurrence plot (RP) is a method to analyze the complexity of a time series, representing the recursion of the phase space trajectory to a certain state (Argyris et al., 1994; Ott, 2002). The construction process of RP has no constraints on mathematical assumptions regarding the data and generation systems (Sun and Wang, 2008). Compared with other nonlinear analysis methods, RP is more suitable for analyzing nonstationary physiological signals. The RQA based on RP is a kind of nonlinear analysis method that quantitatively describes the deterministic structure and complexity of RP according to the distribution of points and line segments in the graph and is suitable for analyzing nonstationary short-term time series such as HRV (Marwan et al., 2002; Marwan et al., 2007).

The R-R interval (RRI) time series was extracted using the Pan-Tompkins algorithm, and automatically correct beats from non-sinus rhythm origin (ectopic beats) by the time-varying threshold method. And the RQA indices were calculated, including the mean diagonal line length (Lmean), maximal diagonal line length (Lmax), recurrence rate (REC), determinism (DET) and Shannon entropy (ShanEn). Both Lmax and Lmean are related to the divergence speed and degree of the phase trajectory, indicating the unpredictability and complexity of the system. REC represents the percentage of recurrence points in RP, which reflects the extent of aggregation of system trajectories in phase space. DET refers to the ratio of recursive points forming a 45° diagonal structure to all recursive points in RP, reflecting the degree of recursion of the orbital period and measuring the certainty (predictability) of the system. ShanEn is used to represent the complexity of the length distribution of diagonal structures in RP.

The quality and effect of the RQA depend on the following three parameters: embedding dimension (m), delay time (τ) and distance threshold (r). According to the experience gained in previous literatures (Takens, 1981; Webber and Zbilut, 1994; Dabiré et al., 1998; Zimatore et al., 2017), the parameters were set to m = 10 and τ = 1, and r was selected to be √m SD (SD is the standard deviation of the RR time series). The above analysis was performed using Kubios HRV Premium software (version 3.1.0, https://www.kubios.com, Kubios Oy, Kuopio, Finland).

### Follow-up

The survival time of LCBM patients was defined from the date of HRV testing to the date of death or the last follow-up. Telephone follow-up was used, and the last follow-up date was 06 March 2022.

### Statistical analysis

The normality of the data was verified by the Kolmogorov-Smirnov test combined with the histogram. There are three main types of data representation: ‾x ± s for normal continuous data, M [Q1, Q3] for non-normal continuous data, and count (percentages) for counting data. For the comparison of all variables between survivors and non-survivors, the unpaired Student’s t-tests and Man-Whitney U test were used to analyze the normal and non-normal continuous data respectively, and chi-square test was used for counting data. The binary classification of RQA indices is carried out by X-Title software, which performs a standard Monte Carlo test to produce a corrected *p* value, thus assessing the statistical significance of data evaluated by multiple cut-offs (Camp et al., 2004). SPSS Statistics 26.0 (IBM Corp., Chicago, Illinois, United States of America) software was used for survival analysis. In the multivariate Cox proportional hazards regression models constructed for each HRV RPA index, the confounding factors with significant significance in univariate analysis were included for adjustment. Survival curves were described using Kaplan–Meier method to estimate the median survival time, and comparisons between survival curves were performed using a log-rank test. Differences were considered to be statistically significant at *p* < 0.05.

## Results

The participants’ general characteristics and comparison of parameters between survived and non-survived groups are shown in Table 1. The mean age and BMI of the patients were 60.4 ± 9.0 years and 22.8 ± 3.3 kg/m^2^, respectively, including 19 females (33.9%) and 37 males (66.1%). Patients with primary tumors of BM are LC, including 40 cases of non-small-cell lung cancer (NSCLC) and 16 cases of small cell lung cancer (SCLC). Extracranial metastasis occurred in twenty-seven patients. The number of patients with multiple BMs accounted for 83.9% (*n* = 47). More than half of the subjects received systemic therapy (n = 32). During our follow-up, with a median follow-up of 19.7 months (range, 1.0–28.7 months), 37 patients died, and 19 patients survived.

**TABLE 1:** Basic characteristics of the LCBM patients enrolled and comparison of parameters between survived and non-survived groups.

	All (N = 56)	Survivors (N = 19)	Non-survivors (N = 37)	*P*
Sex				0.354^a^
Female	19	8	11	
Male	37	11	26	
Age (year)	60.4 ± 9.0	60.1 ± 8.8	60.5 ± 9.2	0.874
BMI (kg/m^2^)	22.8 ± 3.3	24.4 ± 3.1	22.1 ± 3.2	0.012
mean HR (bpm)	79.4 ± 12.9	76.9 ± 13.0	80.7 ± 12.9	0.300
RR (Hz)	0.31 ± 0.07	0.30 ± 0.07	0.32 ± 0.07	0.484
KPS				0.000^a^
≤70	17	0	17	
>70	39	19	20	
Pathological type				0.789^a^
NSCLC	40	14	26	
SCLC	16	5	11	
Extracranial metastasis				0.512^a^
Without	29	11	18	
With	27	8	19	
Primary tumor status				0.625^b^
Not controlled	42	13	29	
Controlled	14	6	8	
Number of BM				0.060^b^
Single	9	6	3	
Multiple	47	13	34	
Systemic treatment after BM				0.935^a^
Without	24	8	16	
With	32	11	21	
Lmean (beats)	15.1 [11.4, 21.4]	15.2 [10.6, 21.2]	15.1 [11.5, 22.6]	0.965
Lmax (beats)	290.2 ± 117.8	265.6 ± 115.8	302.9 ± 118.3	0.265
REC (%)	40.8 ± 12.2	41.9 ± 13.7	40.2 ± 11.5	0.631
DET (%)	98.9 [97.5, 99.5]	98.8 [97.2, 99.6]	98.9 [97.8, 99.5]	0.924
ShanEn	3.5 ± 0.4	3.6 ± 0.5	3.5 ± 0.4	0.493

BMI, body mass index; mean HR, mean heart rate; bpm, beats per minute; RR, respiration rate; KPS, karnofsky performance status; NSCLC, non-small-cell lung cancer; SCLC, small cell lung cancer; BM, brain metastasis; RQA, recurrence quantification analysis; Lmean, mean diagonal line length; Lmax, maximal diagonal line length; REC, recurrence rate; DET, determinism; ShanEn, Shannon entropy; SD: standard deviation; Q1: 1st quartile; Q3: 3rd quartile.

Values are expressed as the number of patients (percentages) or mean ± SD, or median [Q1, Q3].

a The Pearson’s chi squared test.

b The chi square test for continuity correction.

In univariate analysis, the confounding factor, KPS, included in this study was significantly associated with the overall survival (OS) of patients, which mainly shows that the higher KPS was related to higher OS in patients with LCBM (HR = 2.508, 95% CI: 1.307–4.815, *p* = 0.006). There were no significant associations between OS and sex, age, BMI, mean HR, RR, pathological types of LC, primary tumor status, the presence of extracranial metastasis, number of BM or whether systemic therapy was performed after BM in LCBM patients (all with a *p* > 0.05) (Table 2.)

**TABLE 2:** Univariate Cox regression analysis of clinical characteristics and OS in LCBM patients.

	Univariate analysis	
	HR (95% CI)	*P*
Sex		0.750
Female	0.890 (0.434, 1.824)	
Male	Ref	
Age (year)	0.992 (0.956, 1.029)	0.672
BMI (kg/m^2^)	0.912 (0.824, 1.008)	0.073
mean HR (bpm)	1.026 (0.998, 1.055)	0.065
RR (Hz)	42.287 (0.429, 4164.164)	0.110
KPS		0.006
≤70	2.508 (1.307, 4.815)	
>70	Ref	
Pathological types		0.687
NSCLC	1.159 (0.565, 2.376)	
SCLC	Ref	
Primary tumor status		0.597
Not controlled	1.238 (0.562, 2.727)	
Controlled	Ref	
Extracranial metastasis		0.782
Without	0.913 (0.478, 1.743)	
With	Ref	
Number of BM		0.068
Single	0.332(0.102, 1.084)	
Multiple	Ref	
Systemic therapy after BM		0.903
Without	1.042 (0.540, 2.011)	
With	Ref	

BMI, body mass index; mean HR, mean heart rate; bpm, beats per minute; RR, respiration rate; KPS, karnofsky performance status; NSCLC, non-small-cell lung cancer; SCLC, small cell lung cancer; BM, brain metastasis; HR, hazard ratio; CI, confidence interval.

**FIGURE 1:**
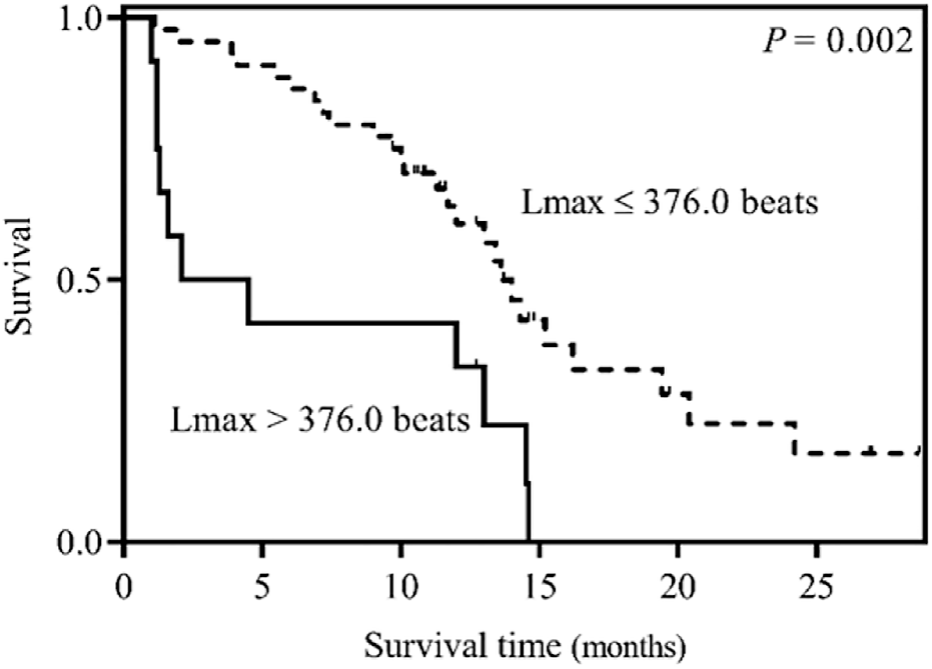
Kaplan–Meier survival curves for LCBM patients stratified by an Lmax of ≤376.0 beats or an Lmax of >376.0 beats.

Univariate analysis of the RQA index showed that the Lmax was significantly correlated with the survival time of LCBM patients. Compared with an Lmax of >376.0 beats group, LCBM patients with an Lmax of ≤376.0 beats group had a longer median survival time (2.1 months vs. 13.6 months, *p* = 0.003) (Table 3 and Figure 1). After adjusting for the confounding factor, KPS, by constructing a multivariate Cox proportional hazard regression model for each the RQA index, the Lmax was still significantly correlated with the survival time of patients with LCBM (HR = 0.318, 95% CI: 0.151–0.669, *p* = 0.003) (Table 3).

**TABLE 3:** Cox regression analysis between HRV and OS in LCBM patients.

	Median survival (M)	Univariate		Multivariate	
		HR (95% CI)	*P*	HR (95% CI)	*P*
Lmean (beats)			0.155		0.408
≤22.5	13.4	0.577 (0.270, 1.232)		0.720 (0.331, 1.568)	
>22.5	13.0	Ref		Ref	
Lmax (beats)			0.003		0.003
≤376.0	13.6	0.329 (0.158, 0.687)		0.318 (0.151, 0.669)	
>376.0	2.1	Ref		Ref	
REC (%)			0.079		0.147
≤42.8	14.3	0.554 (0.287, 1.070)		0.611 (0.315, 1.188)	
>42.8	12.0	Ref		Ref	
DET (%)			0.254		0.139
≤98.2	15.2	0.667 (0.332, 1.337)		0.587 (0.290, 1.189)	
>98.2	12.0	Ref		Ref	
ShanEn			0.249		0.354
≤3.7	12.0	1.562 (0.732, 3.331)		1.433 (0.669, 3.068)	
>3.7	14.0	Ref		Ref	

HR, hazard ratio; CI, confidence interval; Lmean, mean diagonal line length; Lmax, maximal diagonal line length; REC, recurrence rate; DET, determinism; ShanEn, Shannon entropy.

## Discussion

This is a preliminary study to explore the association between nonlinear HRV indices and the survival of LCBM patients. The results showed that whether univariate or multivariate analysis after correction of confounding factors, the RQA index Lmax was related to the prognosis of patients with LCBM.

The three basic mechanisms of tumorigenesis are believed to be controlled by VN via a bidirectional brain-immune pathway in which neurotransmitters are released by a cholinergic anti-inflammatory pathway (Gidron et al., 2005; Zhou et al., 2016). HRV can be used to quantitatively assess the changes in VN activities in an objective and non-invasive manner. Linear HRV analysis has been widely used in cancer prognosis studies. For instance, De Couck et al. (2016) demonstrated that after adjusting for confounding factors, a higher time-domain indicator, SDNN, was associated with longer survival in patients with advanced pancreatic cancer. Wang et al. (2013, 2021) showed a significant correlation between the time-domain index SDNN and the survival time of BM patients, and SDNN was an independent prognostic factor for patients with BM. Wu et al. (2022) first explored the association between HRV time domain indexes (SDNN and RMSSD) and the survival of LCBM patients. The results showed that RMSSD, the time domain index, can be used as an independent prognostic factor for the survival of patients with LCBM. In addition, Chiang et al. (2010) showed that higher frequency-domain index HF in patients with advanced hepatocellular carcinoma was closely related to longer survival time. Chiang et al. (2013) reported that HF was significantly associated with survival of 7 days or less in hospice patients with non-lung cancers. SDNN can reflect the overall variability of HRV, representing the activity of sympathetic and parasympathetic nervous systems, while RMSSD and HF can reflect the vagal activity. Normally, sympathetic and parasympathetic nerves regulate the changes in the heartbeat interval time series through highly complex interactions. Diseases and other causes may lead to ANS dysfunction and alterations in HRV. Although, it has been previously shown that HRV based on linear analysis (time-domain and frequency-domain) may be identified as a prognostic marker for patients with advanced cancers, HRV parameters in the time and frequency domains cannot represent the different transitions between regular, laminar, and chaotic behavior. The existing traditional linear methods for analyzing time series may not be sufficient to evaluate the complexity features of HRV itself (Marwan et al., 2002).

In recent years, the RQA, a nonlinear HRV method based on RP, has been widely used to study the distinction between various disease states and their relative health status and the prediction of disease occurrence. For example, Mohebbi and Ghassemian, (2011) extracted the corresponding Lmax and other RQA indices from the RRIs signal before PAF and distant away from PAF events respectively. The results showed that compared with the ECG segments distant away from PAF, Lmax and other indices in ECG segments before PAF exhibited higher values, and the complexity of the RRIs decreased. For the onset of PAF attacks, features based on the structure of RP have an excellent prediction effect. Acharya et al. (2014) analyzed the heart rate signal of healthy volunteers and patients with CAD using the nonlinear technique. The results revealed that RQA parameters such as the Lmax in CAD subjects were significantly higher compare with the normal subjects. Peng and Sun. (2011) discussed the changes in the complexity of heartbeat interval time series during acute myocardial ischemia using RQA for the first time. The results showed that the complexity decreased during ischemia. Dimitriev et al. (2020) demonstrated that the Lmax exhibited a significant increase (the largest Lyapunov exponent decreased) during mental stress than the rest state, indicating increased predictability and a decreased complexity of the system dynamics during mental stress. All the above studies illustrated that a decrease in the unpredictability and complexity of the heart rate modulation system is closely linked to the disease states of the body.

The Lmax, a complexity measure based on diagonals in RP, is an important recursive variable of the maximum diagonal length except for the main diagonal in RP, and its value is inversely proportional to the Lyapunov exponent (Jp, 1987). The results of this study show that compared with the high-value Lmax group, LCBM patients in the low-value Lmax group had a better prognosis (2.1 months vs. 13.6 months), which we speculate might be related to a theory of complexity loss of the body. The sympathetic and parasympathetic branches of the ANS are competitive in regulating the heart. Therefore, even in the resting state, heartbeat fluctuations of normal humans also show non-stationary and complex characteristics (Goldberger et al., 2002b). As a result, healthy bodies become highly complicated and thus adaptable in the face of various stresses in daily life (Goldberger et al., 2002b; Lipsitz, 2004; McCraty and Shaffer, 2015). However, with the progression of aging, cardiovascular changes occur significantly. Structural factors, such as loss of sinoatrial pacemaker cells or arterial distensibility, and functional changes, such as altered coupling between regulatory components, lead to a decrease in HRV and body complexity; the same phenomenon may also exist in pathophysiological processes (Gribbin et al., 1971; Goldberger, 1996; Pikkujämsä, 1999; Voss et al., 2009). Therefore, the loss of complexity is considered to be a general feature of pathological dynamics to some extent (Javorka et al., 2008). The Lmax is related to the divergence speed and degree of the phase trajectory. A lower Lmax indicates that the phase trajectory segments in the phase space of HRV signal reconstruction are close to each other in a shorter time and diverge quickly; that is, the heart rate regulation system has relatively high unpredictability and complexity. Since the level of Lmax is inversely proportional to the disorder degree of chaos in a dynamic system (Jp, 1987), our results directly support the theory of complexity loss; that is, LCBM patients with poor prognosis have lower complexity of autonomous control.

In addition to Lmax, we also assessed the association between other RQA parameters (Lmean, REC, DET, and ShanEn) and survival in LCBM patients. Lmean measures the unpredictability and complexity of the system. REC reflects the aggregation degree of system trajectories in phase space. DET is a measure of the determinism of the system. ShanEn reflects the complexity of the deterministic structure in the system. Previous studies have shown that although different RQA indexes represent different nonlinear dynamic characteristics, a higher RQA index indicates that the signal changes less, the system changes to low complexity, and the body becomes more and more contrary to the normal state. For example, Mohebbi and Ghassemian. (2011) found that compared with the ECG segments distant away from PAF, Lmean and REC showed higher values in the ECG segments before PAF, and the ShanEn of PAF episodes was significantly higher than that of non-PAF episodes. Acharya et al. (2014) found that the DET and REC were significantly increased in CAD patients compared with normal subjects. However, these four indicators in this study were not statistically significant. There are three possible reasons. First, the sample size is small. Secondly, the measurement time of HRV was before radiotherapy, and emotional tension caused by radiotherapy may affect the HRV. Third, there were no significant differences in age, mean HR, pathological type, extracranial metastasis, primary tumor status and number of BM between survivors and non-survivors. The whole shows a high degree of homogeneity. The above reasons may weaken the prediction ability of RQA parameters.

Our study links nonlinear HRV analysis with cancer prognosis, further expanding previous research on the practical application of RP analysis in the clinical setting and providing new ideas for improving the prognosis of patients with LCBM. It is worth noting that it has been shown that structured aerobic exercise can improve cardiac autonomic nervous modulation in diseased populations (Goldberger et al., 2002a). Therefore, further study is needed to determine whether biofeedback therapy, such as exercise, meditation and music, can improve the autonomic nervous function of patients with LCBM, thereby promoting their treatments.

## Conclusion

In summary, our study demonstrates an independent association between the higher Lmax and poor prognosis in LCBM patients, indicating that the heartbeat complexity characterized by the RQA has a unique contribution to the prognosis of patients with LCBM. Although this study has made progress in finding noninvasive prognostic markers for LCBM patients, there are still some non-negligible limitations. First, the relatively small sample size did not allow us to analyze different LC pathological subgroups, radiotherapy subgroups and radiotherapy dose subgroups. In addition, the measurement time of HRV was before radiotherapy, and emotional tension caused by radiotherapy may impact the HRV of LCBM patients, which cannot be avoided in this study. In conclusion, it is necessary to conduct a prospective study with expanded sample size and a long-term follow-up to continue to evaluate the prognostic role of nonlinear HRV in patients with LCBM, providing more possibilities for patients to choose individualized treatment options.

## Data Availability

The raw data supporting the conclusions of this article will be made available by the authors, without undue reservation.
